# Potassium Hydroxide Impregnated Alumina (KOH-Alumina) as a Recyclable Catalyst for the Solvent-Free Multicomponent Synthesis of Highly Functionalized Substituted Pyridazines and/or Substituted Pyridazin-3(2*H*)-ones under Microwave Irradiation

**DOI:** 10.5402/2011/406427

**Published:** 2011-04-12

**Authors:** Hormi Mecadon, Bekington Myrboh

**Affiliations:** Department of Chemistry, North-Eastern Hill University (NEHU), Permanent Campus, Umshing, Mawlai, Shillong 793022, Meghalaya, India

## Abstract

The work described herein employs potassium hydroxide impregnated alumina (KOH-alumina) as a mild, efficient, and recyclable catalyst for a one-pot solvent-free and environmentally safer synthesis of 3,4,6-triarylpyridazines and some substituted pyridazines from active methylene carbonyl species, 1,2-dicarbonyls, and hydrazine hydrate by microwave (MW) irradiation. The method offers highly convergent, inexpensive, and functionality-tolerable procedure for rapid access to important pyridazine compounds in good yields.

## 1. Introduction

Pyridazines have received considerable attention because of their important pharmacological and biological properties [1]. Several pyridazine compounds exhibit antimicrobial [2], potent analgesic [3], COX inhibitor [4], antidiabetic [5], antihypertensive [6], herbicidal [7], anticancer [8], and antifungal [9] activities. Further, various pyridazinones have been used as intermediates for drugs and agrochemicals [5] and blood platelet aggregation inhibitors [10]. 

The synthesis of pyridazine frameworks has been achieved primarily by the addition of hydrazine or its derivative to an appropriate 1,4-diketones and 1,4-ketoacids [11–13]. Other various pyridazines particularly amino-pyridazines have been prepared from polyfunctionalized nitriles, especially *via* the Jaap-Klingemaan reaction [14–18]. The literature also showed the preparation of pyridazines and pyridazinones involving active methylene species, benzil, and hydrazine. However, the methods employed harsh bases [19–21] or acids [22] in presence of hazardous solvents, and also the reactions require long period of time to complete. Therefore, there is a need for developing a milder and safer solvent-free procedure for the synthesis of substituted pyridazines especially because of the rise in demand for environmentally benign organic synthesis. 

To address the challenge of green synthesis, multicomponent reactions (MCRs) provide a solution since they are more efficient, cost effective, and less wasteful than traditional methods. Such synthetic approach, however, when teamed with microwave (MW) irradiation, facilitates the reaction better as MW gives very efficient thermal management and atom efficiency thus resulting in faster reaction with an increased product yield. In another development, in recent years, the use of inorganic solid supports as catalysts for the synthesis of various biologically active molecules has increased tremendously. Among these inorganic solid supports, potassium hydroxide coated with alumina (KOH-alumina) has been a versatile reagent for various reactions and transformations such as in transesterification and biodiesel production [23–26], ester hydrolysis [27], selective alkylation [28–30], Michael addition [31], cyanoethylation [32], and gas phase dehydrogenations [33]. It has also been found that KOH-alumina exhibited the highest basicity and superior catalytic activity among the alumina-supported alkaline catalysts during transesterification processes [25]. Moreover, KOH-alumina can be prepared easily and is inexpensive. In view of these advantages in the applications of heterogeneous catalysts in the synthesis of heterocyclic compounds, we have chosen KOH-alumina (10% in alumina) for the synthesis of some substituted pyridazines. 

**Scheme 1:**
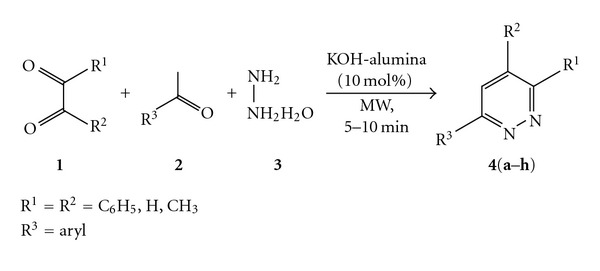
One-pot synthesis of substituted pyridazines.

Therefore, based on our previous work on pyridazine synthesis [21] and in conjunction with our current research aimed at development of synthetic methodologies using solid support catalysts through MCR's [34–36], we report herein the three-component neat synthesis of 3,4,5-triarylpyridazines and other substituted pyridazines using KOH-alumina (10 mol%) by the microwave irradiation technique (Scheme 1).

Initially, the three-component synthesis was optimized by irradiating a mixture of acetophenone (**2a**) (0.2 mL, 1.50 mmol), benzil (**1**) (0.32 g, 1.50 mmol) and hydrazine hydrate (**3**) (0.10 mL, 2.00 mmol) in presence of 5 mol% KOH-alumina in a microwave reactor at 100°C for three minutes which afforded the product **4a** in 57% yield. The same reaction when irradiated for ten minutes gave **4a** in 64% yield. By varying the amount of the catalyst and irradiation time, optimization was finally arrived at 10 mol% of KOH-alumina which significantly resulted in 89% of the product **4a** (Table 1). In another attempt, the catalyst recovered from the reaction after filtration, and washing with ethyl acetate was used further for the condensation of acetophenone (**2a**) (0.20 mL, 1.50 mmol), benzil (**1**) (0.32 g, 1.50 mmol), and hydrazine hydrate (**3**) (0.10 mL, 2.00 mmol). Interestingly, the reaction was observed to complete within 15 min of irradiation giving **4a** in 61% yield. The results of the reactions using recycled KOH-alumina are shown in Table 1.

Thus, the present method was employed for the synthesis of a series of 3,4,5-triarylpyridazines involving different aromatic ketones (**4a**–**g**, Table 2). Irrespective of the presence of different substituents in the *ortho* and *para* positions on the ring of various aromatic aldehydes, the reactions proceeded well to furnish the desired products in good yields (**4a**–**g**, Table 2). Unfortunately, the reaction performed with *meta* substituted aromatic ketones gave only unisolable intermediates and failed to furnish the desired products. On the other hand, polyaromatic acetophenones such as 2-acetylnaphthalene (**2h**) underwent reaction smoothly with benzil (**1**) and hydrazine hydrate (**3**) to afford the desired product **4h **in 77% yield (entry 9, Table 2).

**Scheme 2:**
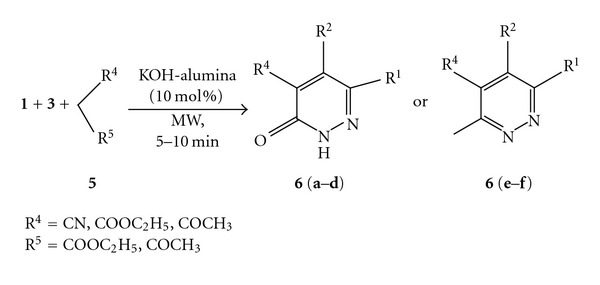
One-pot synthesis of substituted pyridazines.

Similarly, the scope of this methodology was extended to synthesize other substituted pyridazines involving different active methylene carbonyl compounds such as ethyl cyano acetate (**5a**), diethyl malonate (**5b**), ethyl acetoacetate (**5c**), and acetyl acetone (**5d**) (Scheme 2). In all the cases the reactions proceeded fairly well and afforded the desired products in good yields (**6a**–**f**), (Table 3).

The reactions were clean and all the products were purified by simple work-up and crystallization except for products **4d**, **4g**, **4h**, and **6b **which were purified by column chromatography using ethyl acetate and hexane. All the synthesized compounds were characterized by ^1^H NMR, ^13^C NMR, IR, Mass, and Elemental analyses which were found to be in good agreement with the expected data.

**Scheme 3:**
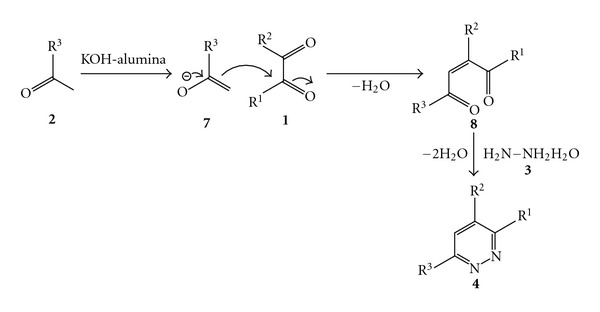
Plausible mechanism for the formation of substituted pyridazines.

From the mechanistic point of view, the formation of the triarlpyridazines **4** probably takes place through the addition of hydrazine hydrate to the 1, 4-dicarbonyl species (**8**) formed *in situ* by reaction between the acetophenone (**2**) and 1,2-dicarbonyl compound (**1**) in a similar fashion as reported earlier [21]. The overall plausible mechanism for the formation of the triarylpyridazines is depicted in Scheme 3.

## 2. Conclusion

In summary, we have established a mild and efficient method for the synthesis of highly functionalized substituted pyridazines and other substituted pyridazinones using KOH-alumina (10 mol%). More importantly, the methodology presented here offers milder, more efficient, and particularly an environmentally friendly approach towards the synthesis of pyridazines by the use of potassium hydroxide impregnated on alumina as a recyclable catalyst.

## 3. Experimental Section

All the chemicals obtained commercially were directly used without further purification. KOH-alumina was prepared according to the procedure reported by Sukata [28], however, as 10% of KOH adsorbed on neutral alumina. Melting points were recorded by open capillary tube method and were uncorrected. The thin layer chromatography was performed on ACME's silica or Merck precoated silica gel and the components were visualized in iodine chamber or by potassium permanganate spray technique. Flash column chromatography was performed on Merck silica gel (60–120 mesh) using ethyl acetate-hexane (3 : 7) as the eluent. IR spectra were recorded with Perkin-Elmer FT-IR spectrometer. The ^1^H and ^13^C NMR were recorded with Bruker AVANCE II 400 FT-NMR machine with TMS as the internal standard. Mass spectra were recorded with Waters ZQ-4000 equipped with ESI and APCI mass detector, and CHN was analyzed on Perkin-Elmer PE 2400 Series II. 

### 3.1. General Procedure

#### 3.1.1. Procedure for the Synthesis of **4(a–h)**

A thoroughly mixed aromatic ketone (**2**) (1.50 mmol), 1,2-dicarbonyl compound (**1**) (1.50 mmol), hydrazine hydrate (**3**) (0.1 mL, 2.00 mmol) in presence of 10 mol% KOH-alumina was irradiated in a Chem Discover microwave reactor at 100°C (power 200 W) at regular intervals of 60 sec for 5–10 min. On completion of the reaction (monitored by thin layer chromatography), the reaction mixture was diluted with ethyl acetate and filtered on a sintered funnel. It was further washed down with ethyl acetate (5 mL × 4). The filtrate was then worked up with cold water, and the organic layer was separated and dried with anhydrous Na_2_SO_4_. The organic filtrate was evaporated in* vacuo* to afford the crude product which was crystallized from ethanol (**4a**, **4b**, **4c**, **4e**, and **4f**) or purified by flash column chromatography (**4d**, **4g**, and **4h**) over silica gel (60–120 mesh) using ethyl acetate-hexane (3 : 7) as the eluent to afford the 3,4,6-triarylpyridazines.

#### 3.1.2. Procedure for the Synthesis of **6(a–f)**

A thoroughly mixed 1,2-dicarbonyl compound (**1**) (1.50 mmol) and hydrazine hydrate (**3**) (2.00 mmol) was irradiated in a Chem Discover microwave reactor at 100°C (power 200 W) for 5 minutes. The mixture was cooled and then introduced therein the active methylene species (**5**) (1.50 mmol) and KOH-alumina (10 mol%). The components were mixed thoroughly and subjected to microwave irradiation at 100°C (power 200 W) for 3–6 minutes. On completion of the reaction (monitored by thin layer chromatography), the reaction mixture was diluted with ethyl acetate and filtered on a sintered funnel. It was further washed down with ethyl acetate (5 mL × 4). The filtrate was then worked up with cold water and the organic layer was separated and dried with anhydrous Na_2_SO_4_. The organic filtrate was evaporated in* vacuo* to afford the crude product which was crystallized from ethanol (**6a**, **6c**, **6d**, **6e**, and** 6f**) or purified by flash column chromatography (**6b**) over silica gel (60–120 mesh) using ethyl acetate-hexane (3 : 7) as the eluent to afford the pure product.

***3,4,6-triphenylpyridazine* (4a, Table 2)**

White solid; mp 182–184°C; ^1^H NMR (400 MHz, CDCl_3_): *δ*_H_ 7.27–7.89 (m, 13H, Ar-H), 8.20 (s, 1H, Ar-H), 8.20 (d, 2H, *J* = 6.8 Hz, Ar-H) ppm; ^13^C NMR (100 MHz, CDCl_3_): *δ*_C_ 124.5, 126.6, 127.6, 128.3, 128.6, 129.5, 129.6, 135.4, 136.0, 136.6, 139.2, 157.2, 157.7 ppm; IR (KBr): *ν*_max⁡_ 1075, 1177, 1394, 1444, 1488, 1582, 2854, 2924, 3063 cm^−1^; MS (ES^+^) for C_22_H_16_N_2_ 308.1 found 308.9 (M + H)^+^, 331.0 (M + Na)^+^; CHN calcd. for C_22_H_16_N_2_ C, 85.69; H, 5.23; N, 9.08 found C, 85.71; H, 5.38; N, 9.32.

***3,4-diphenyl-6-p-tolylpyridazine* (4b, Table 2)**

Light yellow solid; mp 160–162°C; ^1^H NMR (400 MHz, CDCl_3_): *δ*_H_ 2.43 (s, 3H, CH_3_), 7.25–7.35 (m, 10H, Ar-H), 7.48 (d, 2H, *J* = 6.8 Hz, Ar-H), 7.83 (s, 1H, Ar-H), 8.08 (d, 2H, *J* = 8.0 Hz, Ar-H) ppm; ^13^C NMR (100 MHz, CDCl_3_): *δ*_C_ 21.4, 124.7, 126.9, 128.1, 128.7, 129.0, 129.8, 130.0, 133.0, 136.5, 137.1, 139.6, 140.4, 157.6, 157.9 ppm; IR (KBr): *ν*_max⁡_ 1079, 1222, 1367, 1419, 1592, 2867, 2923, 3019 cm^−1^; MS (ES^+^) for C_23_H_18_N_2_ 322.1 found 323.0 (M + H)^+^, 345.0 (M + Na)^+^; CHN calcd. for C_23_H_18_N_2_ C, 85.68; H, 5.63; N, 8.69 found C,85.55; H, 5.65; N, 8.44.

***6-(2-methoxyphenyl)-3,4-diphenylpyridazine* (4c, Table 2)**

White solid; mp 137–139°C; ^1^H NMR (400 MHz, CDCl_3_): *δ*_H_ 3.85 (s, 3H, OCH_3_), 7.00–7.30 (m, 12H, Ar-H), 7.43 (s, 1H, Ar-H), 8.03 (d, 2H, *J* = 7.2 Hz, Ar-H) ppm; ^13^C NMR (100 MHz, CDCl_3_): *δ*_C_ 55.8, 111.4, 121.4, 124.7, 128.2, 128.7, 128.9, 129.2, 130.1, 131.5, 131.6, 136.3, 137.0, 139.1, 156.9, 157.3, 157.7 ppm; IR (KBr): *ν*_max⁡_ 1076, 1199, 1206, 1371, 1496, 1509, 2877, 2943, 3016 cm^−1^; MS (ES^+^) for C_23_H_18_N_2_O 338.1 found 339.0 (M + H)^+^, 361.0 (M + Na)^+^; CHN calcd. for C_23_H_18_N_2_O C, 81.63; H, 5.36; N, 8.28 found C, 81.59; H, 5.16; N, 8.35.

***6-(4-methoxyphenyl)-3,4-diphenylpyridazine* (4d, Table 2)**

Yellow solid; mp 164–166°C; ^1^H NMR (400 MHz, CDCl_3_): *δ*_H_ 3.87 (s, 3H, OCH_3_), 7.04 (d, 2H, *J* = 8.8 Hz, Ar-H), 7.21–7.35 (m, 8H, Ar-H), 7.47 (d, 2H, *J* = 6.8 Hz, Ar-H), 7.79 (s, 1H, Ar-H), 8.15 (d, 2H, *J* = 8.4 Hz, Ar-H) ppm; ^13^C NMR (100 MHz, CDCl_3_): *δ*_C_ 55.39, 114.4, 124.2, 128.2, 128.3, 128.4, 128.6, 128.7, 129.0, 130.0, 136.7, 137.2, 139.5, 157.2, 157.6, 161.3  ppm; IR (KBr): *ν*_max⁡_ 1067, 1208, 1310, 1487, 1562, 2877, 2931, 3012 cm^−1^; MS (ES^+^) for C_23_H_18_N_2_O 338.1 found 339.0 (M + H)^+^, 361.0 (M + Na)^+^; CHN calcd. for C_23_H_18_N_2_O C, 81.63; H, 5.36; N, 8.28 found C, 81.58; H, 5.29; N, 8.11.

***6-(4-bromophenyl)-3,4-diphenylpyridazine* (4e, Table 2)**

White solid; mp 147–149°C; ^1^H NMR (400 MHz, CDCl_3_): *δ*_H_ 7.05–7.45 (m, 12H, Ar-H), 7.74 (s, 1H, Ar-H), 8.08 (d, 2H, *J* = 6.8 Hz) ppm; ^13^C NMR (100 MHz, CDCl_3_): *δ*_C_ 122.6, 125.2, 126.9, 127.2, 128.2, 128.8, 128.9, 129.1, 130.1, 130.2, 131.6, 131.8, 135.8, 136.4, 137.0, 139.9, 157.7, 158.2 ppm; IR (KBr): *ν*_max⁡_ 1075, 1296, 1400, 1488, 1571, 2934, 3011 cm^−1^; MS (ES^+^) for C_22_H_15_BrN_2_ 386.0 found 387.0 (M + H)^+^, 409.0 (M + Na)^+^; CHN calcd. for C_22_H_15_BrN_2_ C, 68.23; H, 3.90; N, 7.23 found C, 68.21; H, 3.73; N, 7.11.

***6-(4-chlorophenyl)-3,4-diphenylpyridazine* (4f, Table 2)**

Light yellow solid; mp 179–181°C; ^1^H NMR (400 MHz, CDCl_3_): *δ*_H_ 7.06–7.44 (m, 12H, Ar-H), 7.73 (s, 1H, Ar-H), 8.08 (d, 2H, *J* = 7.6 Hz) ppm; ^13^C NMR (100 MHz, CDCl_3_): *δ*_C_ 125.1, 126.4, 126.5, 127.1, 127.2, 128.2, 128.6, 128.9, 129.1, 129.5, 129.7, 130.1, 130.2, 135.9, 136.5, 139.8, 157.7, 158.2 ppm; IR (KBr): *ν*_max⁡_ 1076, 1200, 1301, 1481, 1546, 2879, 2932, 3088 cm^−1^; MS (ES^+^) for C_22_H_15_ClN_2_ 342.1 found 343.0 (M + H)^+^, 365.0 (M + Na)^+^; CHN calcd. for C_22_H_15_ClN_2_ C, 77.08; H, 4.41; N, 8.17 found C, 77.26; H, 4.59; N, 8.06.

***6-(4-nitrophenyl)-3,4-diphenylpyridazine* (4g, Table 2)**

White solid; mp 164–166°C; ^1^H NMR (400 MHz, CDCl_3_): *δ*_H_ 7.16–7.44 (m, 11H, Ar-H), 7.56 (t, 1H, *J* = 7.2 Hz, Ar-H), 7.79 (s, 1H, Ar-H), 8.07 (d, 2H, *J* = 8.0 Hz, Ar-H) ppm; ^13^C NMR (100 MHz, CDCl_3_): *δ*_C_ 123.0, 123.6, 126.1, 127.3, 128.2, 128.9, 129.0, 129.1, 129.3, 129.6, 134.4, 135.6, 140.9, 148.1, 157.6, 158.1 ppm; IR (KBr): *ν*_max⁡_ 1076, 1230, 1309, 1441, 1541, 2877, 2932, 3081 cm^−1^; MS (ES^+^) for C_22_H_15_N_3_O_2_ 353.1 found 354.0 (M + H)^+^, 376.0 (M + Na)^+^; CHN calcd. for C_22_H_15_N_3_O_2_ C, 74.78; H, 4.28; N, 11.89 found C, 74.53; H, 4.40; N, 11.76.

***6-(naphthalen-2-yl)-3,4-diphenylpyridazine* (4h, Table 2)**

White solid; mp 190–192°C; ^1^H NMR (400 MHz, CDCl_3_): *δ*_H_ 7.10–7.73 (m, 18H, Ar-H) ppm; ^13^C NMR (100 MHz, CDCl_3_): *δ*_c_ 124.2, 125.4, 125.8, 126.1, 127.3, 127.9, 128.3, 128.8, 129.0, 129.1, 130.2, 130.3, 133.1, 135.6, 136.9, 157.7, 158.2 ppm; IR (KBr): *ν*_max⁡_ 1080, 1234, 1290, 1438, 1521, 2861, 2932, 2995, 3043 cm^−1^; MS (ES^+^) for C_26_H_18_N_2_ 358.1 found 359.0 (M + H)^+^, 381.0 (M + Na)^+^; CHN calcd. for C_26_H_18_N_2_ C, 87.12; H, 5.06; N, 7.82 found C, 87.31; H, 5.21; N, 7.71.

***2,3-dihydro-3-oxo-5,6-diphenylpyridazine-4-carbonitrile* (6a, Table 3)**

White solid; mp 270–272°C; ^1^H NMR (400 MHz, CDCl_3_ + DMSO-*d*_6_): *δ*_H_ 6.91–7.94 (m, 10H, Ar-H), 11.58 (s, 1H, NH) ppm; ^13^C NMR (100 MHz, CDCl_3_ + DMSO-*d*_6_): *δ*_C_ 110.3, 116.5, 127.8, 128.9, 129.1, 129.6, 130.2, 131.4, 132.7, 133.6, 135.3, 146.9, 158.2 ppm; IR (KBr): *ν*_max⁡_ 1010, 1089, 1210, 1464, 1511, 1693, 2256, 2868, 2932, 3412 cm^−1^; MS (ES^+^) for C_17_H_11_N_3_O 273.1 found 274.0 (M + H)^+^, 296.0 (M + Na)^+^; CHN calcd. for C_17_H_11_N_3_O C, 74.71; H, 4.06; N, 15.38 found C, 74.86; H, 4.21; N, 15.12.

***2,3-dihydro-3-oxopyridazine-4-carbonitrile* (6b, Table 3)**

White solid; mp 182–184°C; ^1^H NMR (400 MHz, CDCl_3_ + DMSO-*d*_6_): *δ*_H_ 7.24 (s, 1H, Ar-H), 7.63 (s, 1H, Ar-H), 11.57 (s, 1H, NH) ppm; ^13^C NMR (100 MHz, CDCl_3_ + DMSO-*d*_6_): *δ*_C_ 110.4, 118.3, 138.7, 152.3, 168.8 ppm; IR (KBr): *ν*_max⁡_ 1089, 1212, 1400, 1526, 1676, 2247, 2881, 2932, 3310 cm^−1^; MS (ES^+^) for C_5_H_3_N_3_O 121.0 found 122.0 (M + H)^+^, 144.0 (M + Na)^+^; CHN calcd. for C_5_H_3_N_3_O C, 49.59; H, 2.50; N, 34.70 found C, 49.53; H, 2.41; N, 34.62.

***2,3-dihydro-5,6-dimethyl-3-oxopyridazine-4-carbonitrile* (6c, Table 3)**

White solid; mp 209–211°C; ^1^H NMR (400 MHz, CDCl_3_ + DMSO-*d*_6_): *δ*_H_ 2.33 (s, 3H, CH_3_), 2.50 (s, 3H, CH_3_), 11.25 (s, 1H, NH) ppm; ^13^C NMR (100 MHz, CDCl_3_ + DMSO-*d*_6_): *δ*_C_ 9.9, 27.6, 116.7, 126.2, 152.8, 157.4, 167.9 ppm; IR (KBr): *ν*_max⁡_ 1046, 1287, 1412, 1547, 1671, 2219, 2931, 3349 cm^−1^; MS (ES^+^) for C_7_H_7_N_3_O 149.1 found 150.0 (M + H)^+^, 172.0 (M + Na)^+^; CHN calcd. for C_7_H_7_N_3_O C, 56.37; H, 4.73; N, 28.17 found C, 56.21; H, 4.68; N, 28.32.

***Ethyl 2,3-dihydro-3-oxo-5,6-diphenylpyridazine-4-carboxylate* (6d, Table 3)**

White solid; mp 217–219°C; ^1^H NMR (400 MHz, CDCl_3_): *δ*_H_ 0.90 (t, 3H, *J* = 7.2 Hz, CH_3_), 4.05 (q, 2H, *J* = 7.2 Hz, CH_2_), 7.03–7.27 (m, 10H, Ar-H), 12.56 (s, 1H, NH) ppm; ^13^C NMR (100 MHz, CDCl_3_): *δ*_C_ 13.7, 62.0, 128.0, 128.3, 128.7, 129.1, 129.2, 133.7, 133.8, 134.7, 143.3, 147.7, 158.7, 163.6 ppm; IR (KBr): *ν*_max⁡_ 1101, 1200, 1432, 1500, 1672, 1768, 2867, 2931, 3401 cm^−1^; MS (ES^+^) for C_19_H_16_N_2_O_3_ 320.1 found 321.0 (M + H)^+^, 343.0 (M + Na)^+^; CHN calcd. for C_19_H_16_N_2_O_3_ C, 71.24; H, 5.03; N, 8.74 found C, 71.43; H, 5.17; N, 8.68.

***Ethyl 3-methyl-5,6-diphenylpyridazine-4-carboxylate* (6e, Table 3)**

White solid; mp 77–79°C; ^1^H NMR (400 MHz, CDCl_3_ + DMSO-*d*_6_): *δ*_H_ 0.95 (t, 3H, *J* = 7.4 Hz, CH_3_), 2.65 (s, 3H, CH_3_), 4.05 (q, 2H, *J* = 7.6 Hz, CH_2_), 7.14–7.46 (m, 10H, Ar-H) ppm; ^13^C NMR (100 MHz, CDCl_3_ + DMSO-*d*_6_): *δ*_C_ 14.6, 23.7, 61.4, 126.7, 126.9, 127.5, 128.3, 128.8, 129.3, 130.1, 132.1, 132.7, 134.6, 137.8, 139.5, 141.0, 152.6, 196.3 ppm; IR (KBr): *ν*_max⁡_ 1087, 1100, 1280, 1434, 1510, 1769, 2862, 2932, 3084 cm^−1^; MS (ES^+^) for C_20_H_18_N_2_O_2_ 318.1 found 319.0 (M + H)^+^, 341.0 (M + Na)^+^; CHN calcd. for C_20_H_18_N_2_O_2_ C, 75.45; H, 5.70; N, 8.80 found C, 75.57; H, 5.69; N, 8.82.

***1-(3-methyl-5,6-diphenylpyridazin-4-yl)ethanone* (6f, Table 3)**

White solid; mp 132–134°C; ^1^H NMR (400 MHz, CDCl_3_): *δ*_H_ 1.82 (s, 3H, CH_3_), 2.59 (s, 3H, CH_3_), 7.18–7.47 (m, 10H, Ar-H) ppm; ^13^C NMR (100 MHz, CDCl_3_): *δ*_C_ 21.9, 27.6, 126.7, 126.8, 128.5, 129.3, 129.7, 130.0, 134.6, 135.8, 136.6, 138.4, 138.8, 151.2, 152.3, 186.9 ppm; IR (KBr): *ν*_max⁡_ 1201, 1240, 1433, 1520, 1692, 2888, 2932, 3100 cm^−1^; MS (ES^+^) for C_19_H_16_N_2_O 288.1 found 289.0 (M + H)^+^, 311.0 (M + Na)^+^; CHN calcd. for C_19_H_16_N_2_O C, 79.14; H, 5.59; N, 9.72 found C, 79.33; H, 5.51; N, 9.77.

## Figures and Tables

**Table 1:** Optimization of the reaction condition and the catalyst recyclability with compound **4a**.

Entry	Reaction conditions	Time (min)	Yield (%)^a^
1	KOH-alumina (5 mol%)	3	57
2	KOH-alumina (5 mol%)	10	64
3	KOH-alumina (10 mol%)	2	60
4	KOH-alumina (10 mol%)	4	72
5	KOH-alumina (10 mol%)	6	89
6	KOH-alumina (10 mol%)	8	88
7	KOH-alumina (recycled once)	15	61
8	KOH-alumina (recycled twice)	18	30
9	KOH-alumina (recycled three times)	22	27

^a^Isolated yields.

**Table 2:** KOH-alumina (10 mol%) catalyzed solvent-free synthesis of substituted pyridazines under microwave (MW) irradiation.

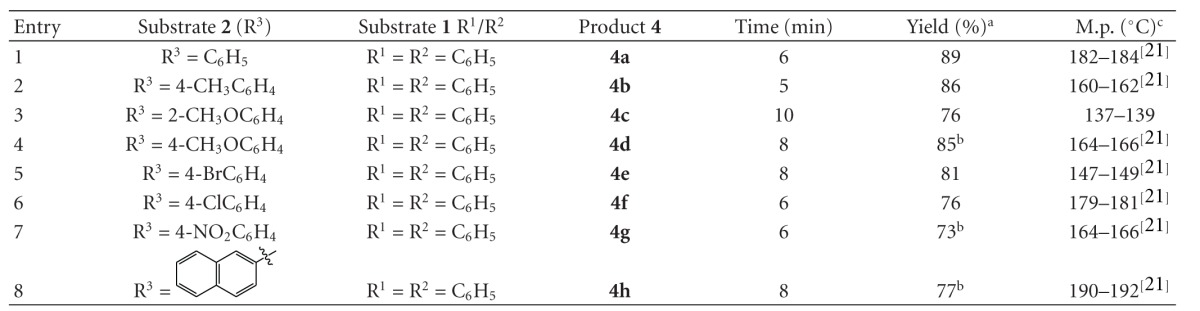

^a^Solated yield. ^b^Purified by column chromatography. ^c^Literature references.

**Table 3:** KOH-alumina (10 mol%) catalyzed solvent-free synthesis of substituted pyridazines under microwave (MW) irradiation.

Entry	Substrate **5 **(R^4^, R^5^)	Substrate **1** R^1^/R^2^	Product **6**	Time (min)	Yield (%)^a^	M.p. (°C)^c^
1	R^4^ = CN,	R^1^ = R^2^ = C_6_H_5_	**6a**	8	81	270–272^[19]^
	R^5^ = COOC_2_H_5_ (**5a**)					
2	(**5a**)	R^1^ = R^2^ = H	**6b**	8	76^b^	182–184^[19]^
3	(**5a**)	R^1^ = R^2^ = CH_3_	**6c**	8	87	209–211^[19]^
4	R^4^ = R^5^ = COOC_2_H_5_ (**5b**)	R^1^ = R^2^ = C_6_H_5_	**6d**	8	82	217–219^[19]^
5	R^4^ = COOC_2_H_5_,	R^1^ = R^2^ = C_6_H_5_	**6e**	8	86	77–79^[20]^
	R^5^ = COCH_3_ (**5c**)					
6	R^4^ = R^5^ = COCH_3_ (**5d**)	R^1^ = R^2^ = C_6_H_5_	**6f**	8	79	132–134^[20]^

^a^Isolated yield. ^b^Purified by column chromatography. ^c^Literature references.
